# Diagnostic Precision in Pediatric Neuromuscular Disorders: A Case Study of Bethlem Myopathy Mimicking Duchenne Muscular Dystrophy

**DOI:** 10.7759/cureus.97510

**Published:** 2025-11-22

**Authors:** Nasher H Alyami, Sarah H Musallam, Hasan Al Greshah, Nasser Alabatahee, Taghreed Alyami, Anwar Al Abdali

**Affiliations:** 1 Laboratory Medicine Department, Hematology Unit, Najran General Hospital, Najran, SAU; 2 Internal Medicine, King Khalid Hospital, Najran, SAU; 3 Hematology, King Khalid Hospital, Najran, SAU; 4 Interventional Radiology, Prince Sultan Military Medical City, Riyadh, SAU; 5 Radiology, King Khalid Hospital, Najran, SAU; 6 Laboratory Medicine, West Najran Maternity and Children Hospital, Najran, SAU

**Keywords:** bethlem myopathy, col6a1, duchenne muscular dystrophy, genetic diagnosis, muscular dystrophy, pediatric myopathy, splice site mutation, whole exome sequencing

## Abstract

Duchenne muscular dystrophy (DMD) is the most prevalent and severe form of childhood muscular dystrophy, typically diagnosed in male children who present with progressive proximal muscle weakness, elevated serum creatine kinase (CK), and delayed motor milestones. Phenotypic overlaps with rarer congenital myopathies, however, can complicate early diagnosis. This report describes a four-year-old male who exhibited classic DMD features, including difficulty rising from the floor (Gowers' sign), calf pseudohypertrophy, generalized hypotonia, and a CK level of 1,200 IU/L. Initial multiplex ligation-dependent probe amplification (MLPA) testing for DMD gene deletions and duplications was negative. Whole exome sequencing (WES) subsequently identified a heterozygous pathogenic splice-site variant (c.1056+1G>A) in the COL6A1 gene, confirming a diagnosis of Bethlem myopathy type 1, an autosomal dominant collagen VI-related disorder. The patient was enrolled in a multidisciplinary care program including physical therapy focused on preserving joint mobility and muscle strength, as well as nutritional support to address failure to thrive. This case illustrates the diagnostic challenges posed by phenotypic similarities between dystrophinopathies and collagen myopathies and emphasizes the essential role of WES when first-line genetic testing is inconclusive. Accurate molecular diagnosis informs prognosis, multidisciplinary management, and genetic counseling, thereby enhancing patient care.

## Introduction

Duchenne muscular dystrophy (DMD) is widely regarded as the prototypical severe pediatric muscular dystrophy, with an incidence of approximately 1 in 3,500 to 5,000 male births. The clinical course is marked by progressive proximal muscle weakness, Gowers’ maneuver, calf pseudohypertrophy, and significantly elevated serum creatine kinase (CK) levels, typically resulting in loss of independent ambulation during early adolescence [1]. Owing to the distinctiveness of this phenotype, DMD is often the initial diagnostic consideration in young males presenting with these features [1,2]. The primary molecular diagnostic technique, multiplex ligation-dependent probe amplification (MLPA), targeting the DMD gene, detects deletions or duplications that account for up to 70% of DMD cases [3]. Nevertheless, this method is restricted to the most common mutations in a single gene, leaving a substantial proportion of cases unresolved and failing to exclude other genetic myopathies with overlapping clinical presentations.

Diagnostic uncertainty is compounded by the presence of DMD phenocopies, such as collagen VI-related dystrophies (COL6-RD), which collectively have a prevalence of 0.6-1 per 100,000 live births [4]. Among these, Bethlem myopathy type 1A (BTHLM1A) constitutes the mildest phenotype and is typically defined by slowly progressive muscle weakness, joint contractures, and occasional skin involvement [4,5]. In early childhood, BTHLM1A may closely resemble DMD, presenting with hypotonia, proximal weakness, calf pseudohypertrophy, and hyperCKemia, while the classic hallmark of distal contractures can be notably absent and often emerges only later in the disease course [6].

Advances in next-generation sequencing (NGS), particularly whole-exome sequencing (WES), now allow for the simultaneous analysis of all known myopathy-associated genes, facilitating definitive molecular diagnosis even in phenotypically ambiguous or complex cases [7].

Here, we present a pediatric case clinically suspected of having DMD who was ultimately diagnosed with BTHLM1A following WES identification of a pathogenic splice site variant within COL6A1. This case underscores the importance of comprehensive genetic testing in muscular dystrophies with overlapping clinical presentations.

## Case presentation

**Figure 1:**
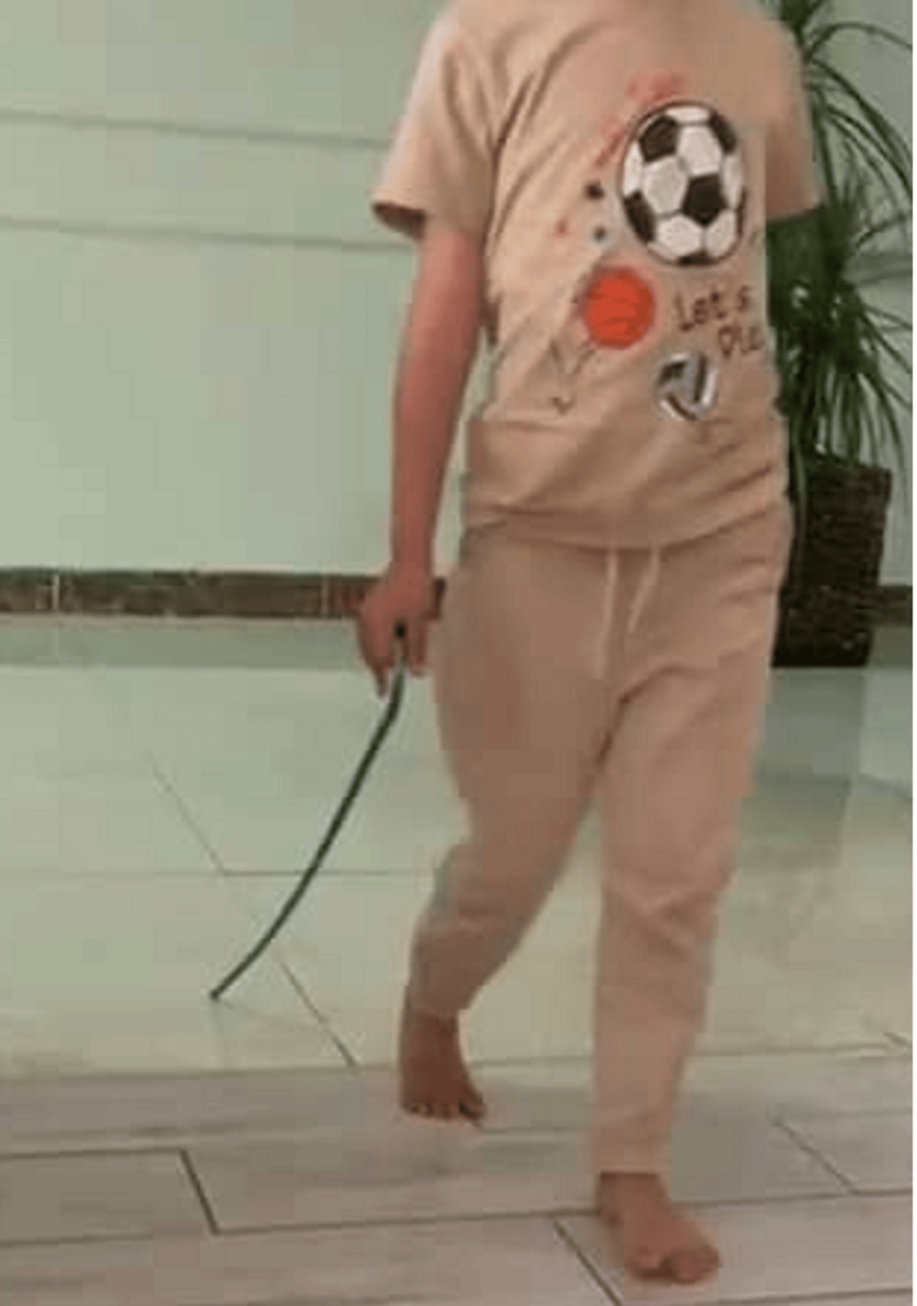
Patient ambulating with a walker due to proximal muscle weakness and mild lower limb contractures typical of early Bethlem myopathy.

A four-year-old male was referred to a tertiary neuromuscular clinic for significant delays in gross motor milestones and a presumptive diagnosis of Duchenne muscular dystrophy. There was no significant family history of neuromuscular disease. Developmental milestones included sitting at 10 months, crawling at 12 months, and independent walking at 20 months. From the onset of ambulation, he exhibited a classic Gowers' maneuver (characterized by pushing on his thighs to stand) and was never able to run (Figure 1). Parents noted progressive calf muscle enlargement from infancy, poor weight gain, and intermittent joint pain.

Physical examination revealed a weight of 19 kg (<5th percentile) and a height of 118 cm (10th percentile), indicating failure to thrive. Bilateral calf pseudohypertrophy was striking. Manual muscle testing showed symmetric proximal weakness (MRC 4/5 at hip flexors and shoulder abductors), with preserved distal strength. Hypotonia and globally reduced deep tendon reflexes were present. Joint tenderness and decreased range of motion at the knees and ankles were noted, but fixed contractures were absent, an atypical feature for Bethlem myopathy, yet one that fits within its early phenotypic spectrum.

The initial diagnostic evaluation demonstrated a markedly elevated serum creatine kinase (CK) level of 1,200 IU/L (reference range: 30-200 IU/L), consistent with active myopathy. A complete blood count revealed mild, non-specific haematological abnormalities (Table 1).

**Table 1:** Initial laboratory investigations at presentation. CK: Creatine Kinase; MCV: Mean Corpuscular Volume

Test	Patient Value	Reference Range	Units
Creatine Kinase (CK)	1,200	30 - 200	international units per liter
Complete Blood Count			
Hemoglobin	12.5	11.5 - 14.5	grams per deciliter
Mean Corpuscular Volume (MCV)	73.4	75 - 87	femtoliters
Platelet Count	356	150 - 450	x 10³ per microliter
Neutrophils	27.4	30 - 45	percent
Absolute Lymphocyte Count	2.99	3.0 - 9.0	x 10³ per microliter

Due to a strong clinical suspicion of Duchenne muscular dystrophy (DMD), first-line genetic testing was performed using multiplex ligation-dependent probe amplification (MLPA) of the DMD gene, which was negative for deletions or duplications.

**Figure 2:**
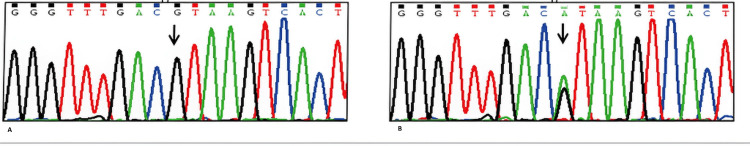
Sequence analysis results showing the heterozygous autosomal dominant variant c.1056+1G>A at the splice donor site at the beginning of intron 14 in the COL6A1 gene. The nucleotide change c.1056+1G>A is indicated by a black arrow. Panel A represents the wild-type allele, while panel B represents the mutated allele.

To address the diagnostic uncertainty, proband-only whole exome sequencing (WES) identified a heterozygous canonical splice-site variant (c.1056+1G>A) in COL6A1 (NM_001848.3), absent from population databases including the Genome Aggregation Database (gnomAD). This variant disrupts the donor splice site of exon 14 and is classified as pathogenic per American College of Medical Genetics and Genomics (ACMG) guidelines (criteria PVS1, PM2) [8], confirming Bethlem myopathy type 1A (Figure 2). Given the definitive molecular diagnosis, muscle biopsy was deferred.

**Figure 3:**
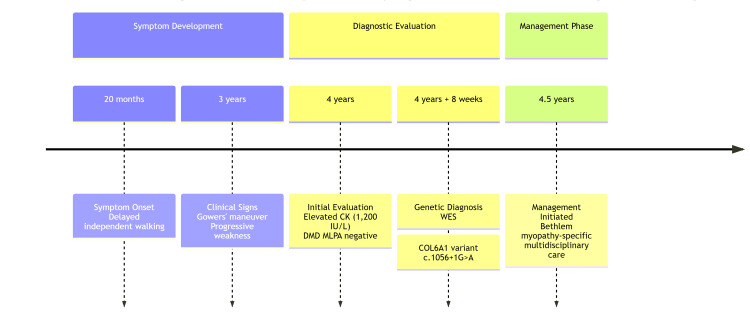
Diagnostic timeline illustrating the stepwise evaluation process. This timeline depicts the sequential diagnostic journey from initial symptom presentation to final molecular diagnosis and execution of condition-specific treatment. The process lasted from 20 months to 4.5 years of age, highlighting the importance of successive testing when phenotypic overlap obscures the underlying cause. WES: whole exome sequencing; MLPA: multiplex ligation-dependent probe amplification.

The timeline outlines the systematic diagnostic journey from initial symptom presentation to definitive molecular diagnosis and implementation of condition-specific management, which are mentioned in Figure 3. 

After diagnosis, the patient was enrolled in a multidisciplinary management program that included physical therapy to maintain joint mobility and muscle strength, as well as nutritional support to address failure to thrive. Genetic counseling was provided to the family, and parental segregation testing is ongoing to determine the inheritance pattern. At the six-month follow-up, the patient's muscle strength and functional status remained stable, indicating a favorable initial response to the supportive care regimen.

## Discussion

This case underscores the diagnostic complexities inherent in pediatric neuromuscular disorders, where phenotypic overlap often masks the true etiology. The patient presented with a classic triad of proximal muscle weakness, calf pseudohypertrophy, and a markedly elevated serum creatine kinase (CK) level, a clinical picture highly suggestive of Duchenne muscular dystrophy (DMD). The absence of pathogenic variants in the DMD gene via multiplex ligation-dependent probe amplification (MLPA) therefore mandated a broader diagnostic strategy. Whole exome sequencing (WES) was pivotal in identifying a heterozygous pathogenic splice-site variant in COL6A1 (NM_001848.3:c.1056+1G>A), leading to a revised diagnosis of Bethlem myopathy type 1 (BTHLM1), an autosomal dominant collagen VI-related disorder [5,8].

This diagnostic odyssey reflects a well-documented challenge in the literature, where Bethlem myopathy can closely mimic limb-girdle muscular dystrophy (LGMD) when proximal weakness predominates [5,9]. Several features of this case deviated from the classic Bethlem myopathy phenotype, complicating the initial assessment. The observed creatine kinase level of 1,200 IU/L was slightly higher than the mild hyperCKemia (typically reported as less than 1,000 IU/L) that is characteristic of this disorder, a finding that initially steered the diagnostic consideration toward other myopathies such as sarcoglycanopathies [9,10]. Furthermore, fixed joint contractures, a well-established hallmark of the disease, were absent upon initial evaluation. It is recognized, however, that these contractures may not be present in early childhood and often develop later in the disease course [11]. These observations underscore the considerable phenotypic heterogeneity in collagen VI-related myopathies and highlight the limitations of relying solely on classic clinical and laboratory features for an early diagnosis.

The phenotypic spectrum of collagen VI-related disorders is broad, ranging from severe Ullrich congenital muscular dystrophy (UCMD) to the milder Bethlem myopathy [12]. The identified COL6A1 variant, located within the critical triple-helical domain, has been previously associated with intermediate phenotypes [13]. This observation aligns with established genotype-phenotype correlations, wherein splice-site mutations often lead to in-frame exon skipping and a clinical course of slowly progressive weakness, contractures, and distal hyperlaxity, typically with preserved ambulation into adulthood [13,14]. This prognosis contrasts starkly with the rapid deterioration characteristic of DMD, underscoring the critical importance of a definitive molecular diagnosis for accurate prognostication and family counseling [14,15].

The lack of a relevant family history in this case suggests a de novo mutation, a recognized mechanism in autosomal dominant collagen VI disorders. This finding emphasizes the necessity of genetic counseling and parental segregation studies to delineate inheritance patterns and recurrence risks [12]. The diagnostic pathway in this patient also illustrates the evolving paradigm in neuromuscular medicine. While muscle biopsy was historically a cornerstone of diagnosis, its utility in Bethlem myopathy is limited by nonspecific findings and often normal collagen VI immunostaining [16]. Consequently, it is no longer recommended as a first-line investigation. Instead, WES has emerged as an efficient and powerful primary diagnostic tool, capable of providing a definitive diagnosis, guiding management, and obviating the need for invasive procedures.

The accurate differentiation of BTHLM1 from DMD carries profound implications for clinical management. The approach shifts from the anticipatory, aggressive cardiopulmonary monitoring required in DMD to a long-term, multidisciplinary strategy focused on preserving joint mobility through physical therapy and surveilling for the insidious onset of respiratory compromise that can occur in collagen VI myopathies [15]. This case affirms that WES should be integrated early in the diagnostic algorithm for complex pediatric myopathies.

A limitation of this report is the absence of histopathological correlation and the relatively short clinical follow-up period, which precludes insights into the long-term disease trajectory.

## Conclusions

This case highlights the diagnostic challenges posed by phenotypic overlap between DMD and other congenital myopathies in early childhood. It underscores the limitations of targeted testing and the essential role of comprehensive genomic analysis in achieving a definitive diagnosis. Accurate molecular identification of Bethlem myopathy type 1A in a child initially presumed to have DMD directly informed prognosis, management, and genetic counseling. Increasing awareness of DMD mimickers and early adoption of broad genetic testing can avoid misdiagnosis, improve care, and ultimately enhance outcomes for children with neuromuscular disorders.

## References

[1] Venugopal V, Pavlakis S (2023). Duchenne Muscular Dystrophy. https://pubmed.ncbi.nlm.nih.gov/29493971/.

[2] Orozco M, Kestler E, Ramirez G (2025). Genetic and clinical landscape of Duchenne muscular dystrophy in Guatemala: insights from a national study. Front Genet.

[3] Dunn DM, Weiss RB (2026). Multiplex ligation-dependent probe amplification (MLPA) for the detection of copy number mutations in the DMD gene. Methods Mol Biol.

[4] Saroja AO, Naik KR, Nalini A, Gayathri N (2013). Bethlem myopathy: An autosomal dominant myopathy with flexion contractures, keloids, and follicular hyperkeratosis. Ann Indian Acad Neurol.

[5] Martins AI, Maarque C, Pinto-Basto J, Negrão L (2017). Bethlem myopathy in a Portuguese patient - case report. Acta Myol.

[6] Bao M, Mao F, Zhao Z (2019). COL6A1 mutation leading to Bethlem myopathy with recurrent hematuria: a case report. BMC Neurol.

[7] de Feraudy Y, Vandroux M, Romero NB (2024). Exome sequencing in undiagnosed congenital myopathy reveals new genes and refines genes-phenotypes correlations. Genome Med.

[8] Hicks D, Lampe AK, Barresi R (2008). A refined diagnostic algorithm for Bethlem myopathy. Neurology.

[9] Mahmood OA, Jiang XM (2014). Limb-girdle muscular dystrophies: where next after six decades from the first proposal (Review). Mol Med Rep.

[10] Panadés-de Oliveira L, Rodríguez-López C, Cantero Montenegro D (2019). Bethlem myopathy: a series of 16 patients and description of seven new associated mutations. J Neurol.

[11] Aldharee H, Hamdan HZ (2024). Segregation of the COL6A2 variant (c.1817-3C>G) in a consanguineous Saudi family with Bethlem myopathy. Genes (Basel).

[12] Bönnemann CG (2011). The collagen VI-related myopathies Ullrich congenital muscular dystrophy and Bethlem myopathy. Handb Clin Neurol.

[13] Pan TC, Zhang RZ, Sudano DG (2003). New molecular mechanism for Ullrich congenital muscular dystrophy: a heterozygous in-frame deletion in the COL6A1 gene causes a severe phenotype. Am J Hum Genet.

[14] Hu C, Shi Y, Zhao L (2024). Clinical, pathologic, and genetic spectrum of collagen VI-related disorder in China-a retrospective observational multicenter study. Hum Mutat.

[15] Foley AR, Quijano-Roy S, Collins J (2013). Natural history of pulmonary function in collagen VI-related myopathies. Brain.

[16] Bönnemann CG, Wang CH, Quijano-Roy S (2014). Diagnostic approach to the congenital muscular dystrophies. Neuromuscul Disord.

